# Dr. Knowles, on Vaccination

**Published:** 1806-10

**Authors:** J. S. Knowles

**Affiliations:** Northumberland Street


### *To the Editors of the Medical and Phyſical Journal.*

Gentlemen,

It must be grateful to the feelings of every well-wisher to his country, to find an object most interesting to its welfare welfare adopted with zeal and spirit by government.— Vaccination is a discovery of no common importance; it guards the life of the subject, and therefore well deserves the patronage of our rulers.

We cannot be surprised that there should have been a certain wariness and hesitation in adopting so new and so important a practice; and we must suppose its progress would be retarded by the timidity of parents, and by the doubts of professional men. It was also a probable circumstance, that a cloud might now and then obscure this dawning blessing, through the ignorance or rashness of many who undertook to disseminate it; but humanity shudders to think that envy and wanton prejudice have been its opposers, and have added forgeries and falsehood to a few disadvantageous occurrences or mistakes, in order to blast its rising reputation.

The consequence has been death to thousands. Now, while the deluded multitude is consigned to the bitterness of grief, the patrons of falsehood triumph in the confusion they have excited. Timely, therefore, is the interference of ministers; and it were to be wished, that vaccination should receive, not only the countenance of government, but likewise obtain its effectual support. Many writers, before me, have remarked, that other countries are deriving the utmost benefit from this discovery, while our own, in which it originated, experiences comparatively little of its salutary effect. Thus it resembles a child, neglected by an unnatural mother, and thriving under the wing of adoption.

This has been justly referred to our liberty, and to the comparative subjection in which other states of Europe are held; so that the privilege which exalts us above the rest of the world is, in this instance, no more than to act like fools and madmen. But if men have a liberty of choice, where evil can result only to themselves, the mischief should, at least, extend no further. If the libeller must not be tolerated, why do we endure him who perverts his talents and influence to the injury of his neighbour?

The College of Physicians, as it has done before, seems likely to decide in favour of Vaccine Inoculation; government will surely then devise the means of giving an effectual check to those who, by disseminating groundless suspicions, and artfully blending truth with falsehood, have contributed to fix a prejudice against vaccination in the minds of their countrymen.

I should,

I should, perhaps, apologize for the strong language in which I have expressed my sentiments respecting the enemies to this practice. It cannot be supposed that any of my observations are applicable to such as may have conceived a doubt, in consequence of reputed failures which, perhaps, originated in the inexperience or incompetency of the inoculator; they are intended only for some malignant spirits, who, unequal to fair discussion, have conjured up pretended proofs, and circumstances innumerable, that never had any real existence, but were the mere phantoms of a dark imagination;—you may refer those gentlemen to a more unfriendly page than mine, the bills of mortality.

I have already said, that upon the introduction of vaccine inoculation, it was not to be expected that it would be universally adopted; it was a question, whether the small-pox would be wholly eradicated, or the people, deluded by a false substitute, be left exposed to its unchecked ravages. Precaution was natural, laudable, and necessary; but the same cause that retarded approbation, should also have suspended an unfavourable decision; inexperience should neither have ensured nor destroyed confidence.

It was likely that mistakes should occur,* where the safety of the practice made every one a Vaccinator, before all the peculiarities of the vaccine disease were ascertained; and who is the man that looks for perfection in medical science without the test of experience?

---

In one of your former Numbers, having observed some discussions respecting a classical title for the Cow-pox, I beg leave to offer you my opinion on that subject. I cannot allow of Bunosus, or Vaccæ-vara, suggested by your correspondent Philologus. In the former there is nothing specific; it might be applied, with equal propriety, to the murrain or the felon. The latter is not a simple or proper appellative, as it consists of two substantives, the one being in the genitive case, and united, not agreeably to the Latin idiom, with the other, which is not even a classical word.† Vaccina being an adjective, cannot be properly employed

* Dr. Jenner has given sufficient precautions in every work he has published. How happy it would have been if practitioners had minutely attended to his directions!

† The term Vara has been introduced into Dictionaries through mistake. Celsus and the two Plinies constantly use the plurals Vari and Varos.

employed as an appellative. Vacciola, and Vacciolation, proposed by Dr. Stokes, are arbitrary terms, not sanctioned by usage. The infinitive, Vaccinare, is now used in the Italian, Spanish, and other languages, derived from the Latin; from this root we must, therefore, form *Vaccinia,* according to the usual analogy of the language; thus, from

| | | |
|---|---|---|
| Lacinare is formed | | Lacinia. |
| Vindicare | ——— | Vindiciæ. |
| Argentare | ——— | Argentaria. |
| Ærare | ——— | Æraria. |
| Lactare | ——— | Lactaria. |

If it be objected, that *Vaccinia* might be confounded with the plural number of Vaccinium, (cow-berry) I answer, that this termination of substantives is frequent in Latin, as well as in the above examples, viz.

Lactarium, *a milk house*; Lactaria, *a milky plant.*
Argentarium, *a piece of plate*; Argentaria, *a silver mine.*
Ærarum, *a treasury*; Æraria, *a copper mine, &c. &c.*

I should have mentioned, that Vaccinatio and Vaccinator are likewise in general use upon the continent as well as in our own islands; so that, I think, we should conform to this usage, and add the term Vaccinia as a classical title for the cow-pox, whether natural or inoculated.

I am, &c.

J. S. KNOWLES.

*Northumberland Street, Aug.* 19, 1806.

